# A Chinese Medicine Formula “Xian-Jia-Tang” for Treating Bladder Outlet Obstruction by Improving Urodynamics and Inhibiting Oxidative Stress through Potassium Channels

**DOI:** 10.1155/2017/8147258

**Published:** 2017-04-19

**Authors:** Jie Sun, Wei Shen, Wenjin An, Qiufen Li, Shunan Qiu, Shaobo Jiang

**Affiliations:** ^1^Department of Urology, The First Affiliated Hospital of Zhejiang Chinese Medical University, Hangzhou City 310006, China; ^2^Department of Nephrology, Hangzhou Hospital of Traditional Chinese Medicine, Hangzhou City 310006, China

## Abstract

The aim of this study is to investigate efficacy of a traditional Chinese medicine formula (named Xian-Jia-Tang, XJT) on bladder outlet obstruction (BOO) in rats and explore its mechanisms. Total 80 BOO model rats were established and randomly divided into 4 groups: physiological saline, XJT, Cesium Chloride (CC), and XJT and CC groups. Meanwhile, 12 rats were used as normal control. Bladder weight and urodynamics were measured. Oxidative stress level and mRNA expressions of potassium channels gene were detected in detrusor. The mRNA and protein levels of hypoxia inducible factor-*α* (HIF-*α*) in detrusor were detected by RT-PCR and Western blot. BOO model rats showed significantly higher bladder weight and abnormal urodynamics. XJT significantly improved the abnormal urodynamics and inhibited the oxidative stress and changes of mRNA levels of potassium channels genes in detrusor of BOO model rats. Moreover,* KATP *and* SK2/3* mRNA were overexpressed in BOO model rats treated by XJT. Besides, the significantly increased levels of HIF-*α* mRNA and protein were also inhibited by XJT. However, these inhibition effects of XJT were weakened by CC. XJT could effectively improve the urodynamics and inhibit the oxidative stress caused by hypoxia through suppressing the role of potassium channels in BOO model rats.

## 1. Introduction

Bladder outlet obstruction (BOO) is a common medical disorder in older males that refers to a blockage that slows or stops urine flow out of the bladder with marked alterations in bladder structure and function, which could result in various lower urinary tract symptoms (LUTS), such as weak urine stream, incomplete urination, frequent urination, and even urge incontinence [1]. BOO is the main consequence of benign prostate hyperplasia (BPH), which is a highly prevalent disease of older men caused by nonmalignant, unregulated growth of the prostate gland [2]. Currently, transurethral resection of the prostate is still the standard treatment of BOO/BPH [3, 4]. However, there were many postoperative complications, such as urge incontinence and sexual dysfunction, which were associated with the decreased quality of life [5, 6]. Thus, it is necessary to find the effective pharmacotherapy for the patients with BOO.

The *α*-blockers and anticholinergic agents are the commonly used drugs for treatment of LUTS, BPH, and BOO at present [7, 8]. Some studies have found the improved quality of life in BOO patients treated by these drugs [9, 10]. However, the application of these drugs was limited by the side effects, such as dizziness, hypotension, and erectile dysfunction [11, 12]. Therefore, new pharmacological strategies on the treatment of BOO are still needed.

Chinese medicine is always considered to be milder and does not have as strong side effects as Western drugs. Xian-Jia-Tang (XJT) is a traditional Chinese medicine formula for invigorating kidney and promoting blood circulation (composition: root of* Rehmannia glutinosa* [shudi], leaves of* Epimedium* [yinyanghuo], fruit of barbary wolfberry [gouqizi], morinda root [bajitian], pangolin scales [chuanshanjia], root of* Salvia miltiorrhiza* [danshen], root of* Rhizoma curcumae* [eshu], and root of szechwan lovage rhizome [chuanxiong] at a rate of 2 : 2 : 3 : 2 : 1 : 2 : 1 : 2 in dry weight, including 2.34 g dry medicine/ml. XJT has been found to be able to suppress the proliferation of prostatic duct epithelium, reduce the deposition of collagen, and inhibit uncoordinated contraction of the bladder, thereby protecting the function of bladder in BPH rats [13]. The shudi, chuanshanjia, danshen, eshu, and chuanxiong can promote blood circulation, and yinyanghuo, gouqizi, and bajitian have an effect of strengthening kidney [13]; the functions of these compositions of XJT may be beneficial to the function of bladder. Many studies have indicated that the danshen and chuanxiong all could stimulate calcium-activated potassium channels in coronary artery smooth muscle cells of pigs and mesenteric artery smooth muscle cells of humans. Meanwhile, it was reported that the BOO was associated with the detrusor overactivity which was associated with the large-conductance calcium-and voltage-activated potassium channels (BK) [14, 15]. Thus, we speculated that the XJT also had efficacy in treating BOO. The mechanism may be associated with the activity of potassium channels in detrusor.

Thus, we performed this study to evaluate the effects of XJT on bladder weight and urodynamics in the BOO model rats. In addition, we also investigated the oxidative stress level and mRNA expression of potassium channels genes in detrusor to explore the mechanisms. The potassium channel inhibitor Cesium Chloride (CC) was used to confirm the role of potassium channels in the mechanism of XJT in treating BOO. In addition, a previous study showed that pharmacological inhibition of HIF pathways is beneficial for the bladder function in BOO model murine [16]. So, we also investigated the mRNA and protein expression of hypoxia inducible factor-*α* (HIF-*α*) in detrusor of BOO model rats and evaluated the effect of XJT on its expression.

## 2. Materials and Methods

### 2.1. BOO Model and Treatment

A total of 108 male Wistar rats (3 months old) were used in this study. Among them, 12 rats were used as normal control and the rest 96 rats were used to establish the BOO model. Bladder outlet was partially obstructed. Simply, these rats were firstly anesthetized with phenobarbital. Abdominal cavity was opened by a midline incision to expose the urethrovesical junction. Then the bladder neck was loosely tied with a 2-0 silk thread to produce partial obstruction in bladder outlet. Finally, the incision was closed and penicillin (0.1 mg/kg) was given by intraperitoneal injection.

One week after surgery, 80 BOO model rats (83.3%) survived. These BOO model rats were randomly divided into 4 groups with 20 rats in each group: in XJT group (*n* = 20), rats were given XJT (1 mL/100 g body weight, each day) by gavage; in CC group, rats were intraperitoneal injected with nonradioactive CC (12 mg/100 g body weight, each day); in XJT + CC group: rats were not only given XJT (1 mL/100 g body weight, each day) by gavage but also injected with CC (12 mg/100 g body weight, each day); and in BOO group, rats were given equal volume physiological saline by gavage. In addition, the 12 rats in normal control group were also treated with equal volume physiological saline. These treatments were continually performed for 30 days. This study was approved by Ethics Committee of the Local Animal Research.

### 2.2. Urodynamic Test

After 30 days of treatment, rats were anesthetized by phenobarbital, and the bladder was exposed by abdominal incision. Then epidural catheter was used as fistula by being inserted into the bladder and fixed with silk. This fistula was connected to a DISA System 2000 urodynamic instrument (Skovlunde, Denmark) and a CMA/100 microinjection pump (Stockholm, Sweden) via a T-tube. Finally, the maximum bladder capacity (MBC) and maximum detrusor pressure (MDP) were measured.

### 2.3. Tissue Sample Preparation

After urodynamic test, the rats were sacrificed by overdose of anesthetic drug. Afterwards, the bladders were rapidly removed and weighed. Then the whole bladders were kept in ice-cold Krebs salt solution until the detrusor was separated from the bladder mucosa by microdissection under sterile conditions. The halves of fresh detrusor muscles in each rat were immediately used for detecting some oxidative stress indices. The rest of detrusor muscles were stored in liquid nitrogen immediately and maintained at −80°C until used for real-time polymerase chain reaction (RT-PCR) or Western blot.

### 2.4. Measurement of Oxidative Stress Indices

Malondialdehyde (MAD) was determined by the thiobarbituric acid assay in which a red-colored complex was produced through the reaction between MAD and thiobarbituric acid. This absorbance of this red-colored complex was measured at 532 nm. The activity of superoxide dismutase (SOD) was measured by quantifying the inhibition of cytochrome-C reduction in xanthine-xanthine oxidase system at 550 nm. The determination of total antioxidative capacity (T-AOC) was measured by using a commercial kit (Nanjing Jiancheng Bioengineering Institute, Nanjing, China) based on the instruction. A UV-2400PC spectrophotometer (Shimadzu, Kyoto, Japan) was used in these measurements.

### 2.5. Real-Time Polymerase Chain Reaction (RT-PCR)

The frozen samples were thawed and homogenized at 4°C in 50 mM Tris-HCl (pH 7.6), using a Teflon homogenizer. Total RNA was extracted using the Trizol reagent (Biobasic, Inc., Ontario, Canada). The cDNA was obtained by reversely transcribing these isolated RNA. Then RT-PCR was performed by a Bio-Rad iCycler (Bio-Rad, Hercules, CA, USA) to determine the mRNA levels of the following genes:* Kv1.5* and* Kv2.1* (encodes voltage-gated potassium channels),* BK *(encodes large-conductance calcium-activated potassium channels),* SK2/3 *(encodes subtypes of small conductance calcium-activated potassium channels), and* KATP *(encodes activates adenosine triphosphate-sensitive potassium channels) as well as HIF-*α* (encodes hypoxia inducible factor-*α*) by using the corresponding primers (Table 1). The reaction program included an initial denaturation at 95°C for 1 min, followed by 40 cycles at 95°C for 10 s and 64°C for 25 s. The mRNA levels of these genes were quantified using 18S rRNA as internal control.

### 2.6. Western Blot

Western blot was performed to detect the expression of HIF-*α* in detrusor. Simply, the frozen samples were thawed and homogenized at 4°C in 50 mM Tris-HCl (pH 7.6), using a Teflon homogenizer. Total proteins were extracted by RIPA Lysis Buffer (Beyotime Institute of Biotechnology, Shanghai) with protease inhibitor cocktail (Sigma, USA). These proteins were separated by sodium dodecyl sulfate-polyacrylamide gel electrophoresis with 10% polyacrylamide gels. Then the proteins were transferred to a polyvinylidene fluoride membrane (Millipore, USA) and blocked with 5% skim milk in Tris buffered saline Tween (10 mm Tris, 15 mm NaCl, 0.05% Tween 20) for 2 h. After incubation with primary antibody (anti-HIF-1, 1 : 500, sc-10790, Santa Cruz) overnight at 4°C, horseradish peroxidase-labeled second antibody (1 : 2000, Santa Cruz) was added and continually incubated for 1 h at 25°C. Finally, the proteins were visualized using enhanced chemiluminescence reagents (Amersham Pharmacia, UK). The density of protein bands was analyzed by Bandscan 5.0 software (Glyko, Novato, CA, USA). The relative level of HIF-*α* was calculated using *β*-actin as internal control.

### 2.7. Statistical Analyses

All data were expressed as mean ± SD. Statistical analysis was performed by SPSS version 17.0 (SPSS Inc., Chicago, IL). Comparison among groups was determined by one-way analysis of variance using least significant difference test (equal variances assumed) or Dunnett's T3 (equal variances not assumed) for post hoc test between groups. A *P* value less than 0.05 was considered as statistically significant.

## 3. Results

### 3.1. Changes of Bladder Weight

As shown in Table 2, the BOO model rats showed significantly lower body weight but higher bladder weight compared with the normal rats (*P* < 0.01). Moreover, the ratio of bladder weight/body weight was significantly higher in the rats of BOO group compared with that in the rats of normal control group (*P* < 0.01). However, the rats in XJT group indicated significantly higher body weight and lower bladder weight and ratio of bladder weight/body weight compared with that in BOO group (*P* < 0.01). Although the bladder weight and ratio of bladder weight/body weight in rats treated by XJT were still significantly higher compared with normal value (*P* < 0.01), the similar body weight was found between XJT and normal control groups (*P* > 0.05). After being injected with CC, the BOO model rats indicated significantly lower body weight and higher ratio of bladder weight/body weight (*P* < 0.01). XJT did not significantly inhibit the decreased body weight of BOO model rats that were injected with CC (*P* > 0.05), while ratio of bladder weight/body weight was significantly decreased in rats of XJT + CC group. The bladder weight was similar among the rats in BOO, CC, and XJT + CC groups (*P* > 0.05). Moreover, the body weight was significantly decreased and bladder weight was significantly increased by CC in XJT + CC group when compared with that in XJT group (*P* < 0.01).

### 3.2. Effect of XJT on Urodynamics of BOO Model Rats

The results showed that the rats in BOO group had significantly higher MDP and lower MBC (*P* < 0.01), indicating urodynamic abnormality. XJT significantly decreased the MDP (*P* < 0.01) and increased the MBC of BOO model rats (*P* = 0.03), which was similar to the value in normal control group (*P* > 0.05). In addition, the significantly higher MDP was also found in the rats of CC group (*P* < 0.01), which was similar to that in the rats of BOO group (*P* > 0.05). Moreover, the mean level of MBC in CC group was significantly lower than that in normal control group (*P* < 0.01) and BOO group (*P* = 0.02). However, XJT could significantly inhibit the increase of MDP (*P* < 0.01) and the decrease of MBC (*P* = 0.03) in XJT + CC group. Moreover, compared with XJT group, CC significantly inhibited the effect of XJT on MDP (*P* = 0.04) and MBC (*P* < 0.01) in XJT + CC group (Table 3).

### 3.3. Effect of XJT on Oxidative Stress in Detrusor

**Figure 1:**
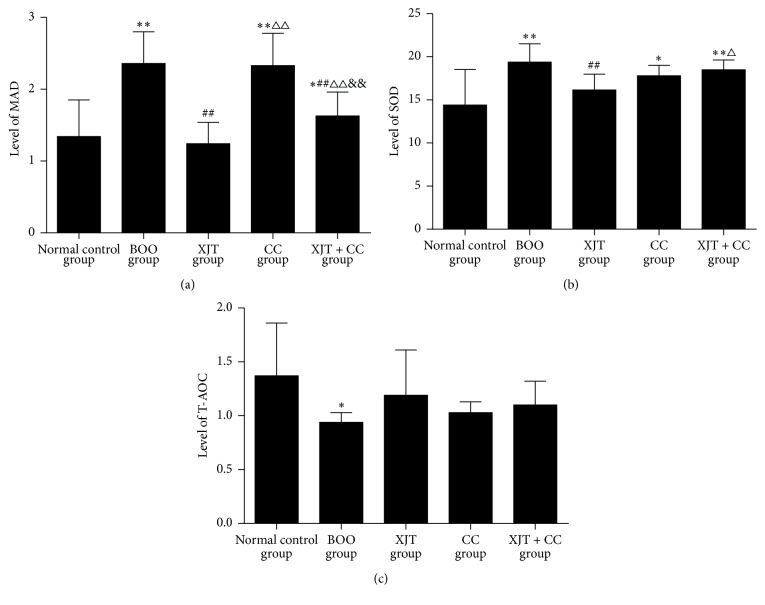
*The levels of MAD (a), SOD (b), and T-AOC (c) in detrusor of rats in each group*. BOO: bladder outlet obstruction; XJT: Xian-Jia-Tang (a traditional Chinese medicine formula for invigorating kidney and promoting blood circulation); CC: Cesium Chloride; MAD: Malondialdehyde; SOD: superoxide dismutase; and T-AOC: total antioxidative capacity. *∗* and △, respectively, represent significant difference compared with normal control and XJT groups, with *P* < 0.05; *∗∗*, ##, △△, and &&, respectively, represent significant difference compared with normal control, BOO, XJT, and CC groups, with *P* < 0.01.

BOO model rats exhibited significantly downregulated level of T-AOC (*P* = 0.01) and upregulated levels of MAD and SOD (*P* < 0.01) when compared with the rats in normal control group. By the intervention of XTJ, the levels of MAD, SOD, and T-AOC were all recovered to normal level (*P* > 0.05). Similar to the BOO group, the levels of MAD (*P* < 0.01) and SOD (*P* = 0.02) were also significantly increased in CC group compared with those in normal control group. Moreover, XJT significantly reduced this increased level of MAD (*P* < 0.01) but did not affect the level of SOD (*P* > 0.05) in XJT + CC group, compared with that in CC group. In addition, results only showed a decreased trend of level of T-AOC in CC group without statistically significant difference (*P* > 0.05) compared with that in normal control group, which was also similar to that in BOO group (*P* > 0.05). Meanwhile, no significantly different levels of T-AOC were found between CC and XJT + CC group (*P* > 0.05). Besides, results also showed the inhibition effects of XJT on the levels of MAD (*P* < 0.01) and SOD (*P* = 0.03) were significantly weakened by CC when comparing XJT + CC group with XJT group (Figure 1).

### 3.4. Effect of XJT on Potassium Channels in Detrusor

As shown in Table 4, the mRNA expressions of* BK*,* SK2/3*, and* KATP* were significantly reduced (*P* < 0.01), while the mRNA expressions of* Kv2.1* and* Kv1.5* were significantly increased (*P* < 0.01) in BOO model rats when compared with normal rats. However, XJT significantly increased the mRNA levels of* BK*,* SK2/3*, and* KATP *(*P* < 0.01) and decreased the mRNA levels of* Kv2.1* (*P* < 0.01) and* Kv1.5 *(*P* = 0.04) in the rats of XJT group compared with those in the rats of BOO group. The level of* BK* mRNA was still lower and the levels of* Kv2.1* and* Kv1.5* mRNA were still higher in XJT group compared with the normal control group (*P* < 0.01), while the levels of* SK2/3* and* KATP* mRNA in XJT even exceeded the normal values (*P* < 0.01). The injection of CC did not significantly affect the levels of* BK*,* SK2/3,* and* Kv1.5* mRNA (*P* > 0.05) but significantly decreased the mRNA level of* Kv2.1* (*P* = 0.02) and increased the mRNA of* KATP* (*P* < 0.01) in BOO model rats. However, XJT significantly decreased the levels of* Kv2.1* and* Kv1.5* mRNA (*P* < 0.05) and increased the level of* KATP *mRNA in XJT + CC group compared with that in CC group (*P* < 0.01) but did not significantly affect the levels of* BK* and* SK2/3 *mRNA (*P* > 0.05). Moreover, compared with XJT group, CC significantly inhibited the effect of XJT on the mRNA levels of* BK*,* SK2/3*,* Kv2.1*, and* KATP* in XJT + CC group (*P* < 0.05).

### 3.5. Effect of XJT on Expression of HIF-*α* mRNA and Protein in Detrusor

**Figure 2:**
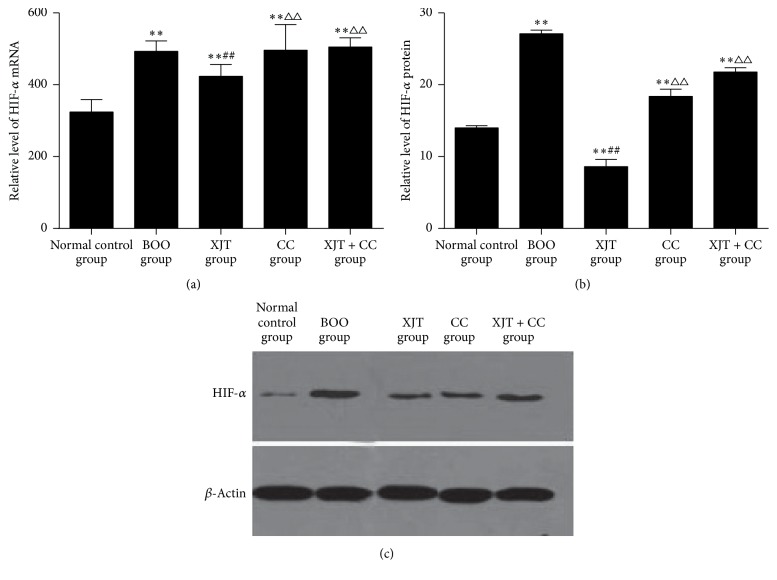
*The relative expression of HIF-α in detrusor of rats in each group*. BOO: bladder outlet obstruction; XJT: Xian-Jia-Tang (a traditional Chinese medicine formula for invigorating kidney and promoting blood circulation); CC: Cesium Chloride; and HIF-*α*: hypoxia inducible factor-*α*. *∗∗*, ##, and △△, respectively, represent significant difference compared with normal control, BOO and XJT groups with *P* < 0.01.

As shown in Figure 2, the mRNA and protein levels of HIF-*α* were significantly increased in BOO model rats compared with those in normal rats (*P* < 0.01). However, the upregulated expressions of HIF-*α* mRNA and protein in BOO model rats were significantly reduced by XJT (*P* < 0.01). Although the level of HIF-*α* mRNA in XJT group was still higher than that in normal control group (*P* < 0.01), the level of HIF-*α* protein was decreased to a lower value by XJT (*P* < 0.01). The levels of HIF-*α* mRNA and protein in CC group were similar to those in BOO group (*P* > 0.05), which were also significantly higher than those in normal control group (*P* < 0.01). In XJT + CC group, XJT did not significantly decrease the higher levels of HIF-*α* mRNA and protein in the BOO model rats that were injected with CC (*P* > 0.05). However, CC significantly affected the inhibition effect of XJT on expression of HIF-*α* mRNA and protein in XJT + CC group when compared with that in XJT group (*P* < 0.01).

## 4. Discussion

In this study, XJT, as a traditional Chinese medicine formula for treatment of BOO without strong side effects as Western drugs, was investigated in rats. Results showed that XJT could significantly inhibit the increased bladder weight and abnormal changes in urodynamics, indicating the role of XJT in preventing bladder damage and protecting bladder function. In addition, results also showed the effect of XJT on the oxidative stress level and potassium channels gene expressions in detrusor of BOO model rats. However, these effects could be significantly weakened by the injection of CC.

BOO could lead to the decrease of blood flow and oxygen tension in the detrusor layer, thereby generating an amount of reactive oxygen species [17, 18]. Some studies have proved the efficacy of antioxidant therapy in the treatment of detrusor dysfunction in BOO animals [19, 20]. In this study, although the effect of XJT on inhibiting the decrease of T-AOC was not statistically significant, the trend is visible. Besides, XJT significantly reduced the increase of MAD and SOD in BOO model rats, which illustrated XJT may protect the detrusor through inhibiting the oxidative stress.

Potassium channels also play important roles in the stability and contractility of detrusor [21]. As reported, downregulation of BK channels may contribute to BOO-induced detrusor overactivity by increasing the phosphorylation level of myosin light chain 2 [14]. Moreover, BK and SK channels were associated with the modulation of human detrusor smooth muscle phasic contractility [22]. The KATP channels were reported to mediate the contractions of detrusor muscles [23]. In this study, the anomalous expressions of potassium channels were found in BOO model rats. XJT significantly inhibited the expressions of* Kv2.1* and* Kv1.5* but increased the expression of* BK*,* SK2/3,* and* KATP* in detrusor. These results indicated that the effect of XJT on BOO was associated with potassium channels in detrusor. Moreover, the overexpression of* KATP* was found in BOO model rats treated with XJT. In addition, results also showed that CC (a potassium channel inhibitor) could significantly weaken the effect of XJT on not only the expression of these potassium channels but also the bladder weight, urodynamics, and oxidative stress. It was reported that the KATP channels were associated with apoptosis induced by oxidative stress [24]. Moreover, the BOO-induced apoptosis in the urinary bladder has been found to be related to the hypoxia-triggered endoplasmic reticulum stress [25], which may be one of the reasons causing the oxidative stress in detrusor of BOO [26]. Thus, XJT may inhibit the oxidative stress induced injury through the activity of KATP channels in BOO model rats.

In addition, results also showed a significantly increased expression of HIF-*α* mRNA and protein in detrusor. Similarly, these increases were significantly inhibited by XJT and CC significantly affected this inhibition effect. As a nuclear transcription factor, HIF-*α* was always highly expressed in anoxic environment, which was an effective marker for hypoxia [27]. In BOO, high pressure in bladder wall always occurred, which could lead to reducing blood supply to the bladder due to oppressed artery [28]. Although subsequent process of urination can relieve the ischemia and hypoxia of bladder tissue, ischemia-reperfusion always enhances the bladder injury [29]. As shown in above discussion, the hypoxia may be the main reason resulting in the oxidative stress in detrusor of BOO [26, 30]. Previous studies have showed that KATP channels showed protective roles in hypoxia induced damages in nerve and vasculature [31, 32]. This evidence proved that KATP channels may play key roles in the inhibition effect of XJT on hypoxia and oxidative stress induced injury in detrusor.

## 5. Conclusions

In conclusion, XJT was found to be effective in the treatment of BOO by inhibiting oxidative stress and increasing the expression of KATP channels in detrusor. The KATP channels may play key roles in protecting the detrusor from oxidative stress induced injury in the BOO model rats treated by XJT. The oxidative stress in detrusor may be caused by hypoxia. However, as a Chinese medicine formula, XJT was composed of a variety of drugs. The active ingredients in XJT and the interaction mechanisms among ingredients should be further explored in further studies.

## Figures and Tables

**Table 1:** Special primers used in RT-PCR.

Gene	Genbank accession	Primer sequences (5′ to 3′)
*Kv1.5*	NM_012972.1	Forward: CTGGGGGTTCCCTGGAGAGTT
		Reverse: GCATACAGGGACCTCCGCAAGT
*Kv2.1*	NM_013186.1	Forward: GAGGAGCACACGGGAGCACTA
		Reverse: GTGGTGGGGGCCTTGGAGTT
*BK*	NM_031828.1	Forward: CGAGGGCCTCACTAAACCATC
		Reverse: GCCGAAGCCGAAAGCGAATA
*KATP*	NM_017023.1	Forward: GGACAACTGTGCTGGACCTGAAA
		Reverse: TCTCCACACAAGGAGTGCGGTT
*SK2/3*	NM_019315.2	Forward: AGGAGTACCAGCGCCAGGTGT
		Reverse: CGGTGAATGGTGCCCGTGA
*HIF-1*	Y09507	Forward: TGCCCGAAAGCTCGAACTCA
		Reverse: GACTTTGGCGTGGTCAATCTTCTT
*18S*	NR_046237	Forward: CGTGCCCCTACTATGTCGCTTT
		Reverse: GTCTTCTGCTCCATTCCATCCTGT

**Table 2:** Bladder weight differences of rats in each group.

Groups	Body weight (g)	Bladder weight (g)	Bladder weight/body weight (‰)
Normal control group	326.25 ± 16.02	0.10 ± 0.01	0.31 ± 0.03
BOO group	303.05 ± 13.14^*∗∗*^	0.43 ± 0.05^*∗∗*^	1.43 ± 0.17^*∗∗*^
XJT group	320.57 ± 35.16^##^	0.33 ± 0.10^*∗∗*##^	1.01 ± 0.30^*∗∗*##^
CC group	265.40 ± 21.81^*∗∗*##△△^	0.47 ± 0.08^*∗∗*△△^	1.77 ± 0.03^*∗∗*##△△^
XJT + CC group	266.30 ± 10.29^*∗∗*##△△^	0.41 ± 0.02^*∗∗*△△^	1.14 ± 0.57^*∗∗*&&^

BOO: bladder outlet obstruction; XJT: Xian-Jia-Tang (a traditional Chinese medicine formula for invigorating kidney and promoting blood circulation); and CC: Cesium Chloride. *∗∗*, ##, △△, and &&, respectively, represent significant difference compared with Normal control, BOO, XJT, and CC groups with *P* < 0.01.

**Table 3:** Changes of urodynamic parameters in the rats of each group.

Groups	MDP (mmH_2_O)	MBC (ml)
Normal control group	106.10 ± 6.91	3.00 ± 0.34
BOO group	160.33 ± 10.37^*∗∗*^	2.35 ± 0.29^*∗∗*^
XJT group	110.29 ± 10.86^##^	2.78 ± 0.30^#^
CC group	156.83 ± 18.87^*∗∗*△△^	1.87 ± 0.29^*∗∗*#△△^
XJT + CC group	129.88 ± 11.60^*∗∗*##△&&^	2.29 ± 0.54^*∗∗*△△&^

BOO: bladder outlet obstruction; XJT: Xian-Jia-Tang (a traditional Chinese medicine formula for invigorating kidney and promoting blood circulation); CC: Cesium Chloride; MBC: maximum bladder capacity; and MDP: maximum detrusor pressure. #, △, and &, respectively, represent significant difference compared with BOO, XJT, and CC groups with *P* < 0.05; *∗∗*, ##, △△, and &&, respectively, represent significant difference compared with Normal control, BOO, XJT, and CC groups with *P* < 0.01.

**Table 4:** The mRNA expression of potassium channels in detrusor of each group.

Groups	*BK*	*SK2/3*	*Kv2.1*	*Kv1.5*	*KATP*
Normal control group	13.38 ± 1.41	16.98 ± 2.28	38.20 ± 8.23	7.98 ± 1.18	8.88 ± 1.59
BOO group	3.30 ± 0.50^*∗∗*^	12.49 ± 1.72^*∗∗*^	93.78 ± 7.83^*∗∗*^	10.99 ± 1.21^*∗∗*^	4.47 ± 1.09^*∗∗*^
XJT group	4.59 ± 0.70^*∗∗*##^	24.23 ± 2.99^*∗∗*##^	59.30 ± 6.66^*∗∗*##^	9.41 ± 1.46^*∗∗*#^	15.32 ± 1.93^*∗∗*##^
CC group	2.66 ± 0.34^*∗∗*△△^	12.68 ± 2.64^*∗∗*△△^	74.23 ± 14.30^*∗∗*#△△^	11.37 ± 1.57^*∗∗*△^	9.19 ± 1.19^##△△^
XJT + CC group	3.13 ± 0.67^*∗∗*△^	11.16 ± 2.70^*∗∗*△△^	63.12 ± 4.62^*∗∗*#△△&^	9.28 ± 0.96^*∗∗*#&^	13.35 ± 2.40^*∗∗*##△&&^

BOO: bladder outlet obstruction; XJT: Xian-Jia-Tang (a traditional Chinese medicine formula for invigorating kidney and promoting blood circulation); and CC: Cesium Chloride. #, △, and &, respectively, represent significant difference compared with BOO, XJT, and CC group with *P* < 0.05; *∗∗*, ##, △△, and &&, respectively, represent significant difference compared with normal control, BOO, XJT, and CC group with *P* < 0.01.

## Acronyms

BOO: Bladder outlet obstruction
LUTS: Lower urinary tract symptoms
BPH: Benign prostate hyperplasia
BK: Large-conductance calcium-and voltage-activated potassium channels
XJT: Xian-Jia-Tang
CC: Cesium Chloride
HIF-*α*: Hypoxia inducible factor-*α*
MBC: Maximum bladder capacity
MDP: Maximum detrusor pressure
RT-PCR: Real-time polymerase chain reaction
MAD: Malondialdehyde
SOD: Superoxide dismutase
T-AOC: Total antioxidative capacity
KATP: Activates adenosine triphosphate-sensitive potassium.
